# The effect of students’ online learning experience on their satisfaction during the COVID-19 pandemic: The mediating role of preference

**DOI:** 10.3389/fpsyg.2023.1095073

**Published:** 2023-01-31

**Authors:** Xinchao Li, Flavian Adhiambo Odhiambo, Dickson Kofi Wiredu Ocansey

**Affiliations:** ^1^Department of Pedagogy, School of Teacher Education, Jiangsu University, Zhenjiang, Jiangsu, China; ^2^Directorate of University Health Services, University of Cape Coast, PMB, Cape Coast, Ghana

**Keywords:** online learning, student satisfaction, preference, student engagement, perceived quality, internet access and cost, self-confidence, COVID-19 pandemic

## Abstract

**Introduction:**

During the peak of the COVID-19 pandemic, nearly all educational institutions globally had to eventually embrace the maneuver of transferring to nearly 100% online learning as a new routine for different curricula. Although many students in developing countries such as Kenya are only experiencing the exclusive online learning approach for the first time, research on students’ experience and satisfaction with COVID-19-imposed online learning is largely lacking. Thus, this study examined the effect of online-learning experiences on satisfaction in the setting of the COVID-19 pandemic in Kenya. The mediating role of students’ preference on the relationship between online-learning experience and satisfaction was also examined.

**Methods:**

A web-based survey involving 501 respondents was analyzed using IBM® SPSS® and AMOS software platforms. A structural equation model (SEM) was used to analyze the relationships.

**Results and Discussion:**

Results showed that 80% of participants indicated their preference for in-person learning as against 20% for online learning. Students’ satisfaction-SS had a significant positive correlation with online classroom perceived quality-OCPQ, acquisition of self-confidence-ASC, teaching performance and engagement-TPE, and preference for online learning-POL but a negative correlation with internet access and cost-IAC. Moreover, while POL positively correlated with OCPQ, ASC, and TPE, it negatively correlated with IAC. Both the structural model for the main effect and the mediation model provided a good fit and confirmed these relationships. Student preference had a significant effect on satisfaction and played a significant mediating role in the relationship between online-learning experience and satisfaction. These findings shed light on the underlying factors that explain students’ online learning satisfaction and provide guidelines for universities and policymakers to make better decisions that enhance students’ online-learning experience and satisfaction.

## 1. Introduction

The media and means through which people embark on learning are fundamentally changing as technology constantly expands, increasing its impacts on the ways people acquire, correct, and update their understanding (Lodge and Harrison, 2019). In effect, new and evolving technologies present immense opportunities for teaching and learning (Szopiński and Bachnik, 2021). The emergence of mobile network devices including cell phones and personalized computers indicates that information can be accessed anywhere and anytime with an Internet connection. This new and constantly evolving information reality carries along a substantial affordance for learning both in informal and formal education settings. In the phase of this development, the world has recently been confronted with a devastating plague, the COVID-19 pandemic, taking a full toll globally in 2020. The COVID-19 situation necessitates embracing new pandemic-imposed conditions, such as distance learning, which need to be addressed by the higher education sector as well. Both students and faculty members were compelled to re-think the application of available technology resources to not only deliver higher education services but also benefit from them as well. However, a critical question remains, as to whether this new setup is effective and satisfies the student’s learning needs.

The COVID-19 pandemic greatly affected all areas of life, including education, as educational institutions were locked down (Khalil et al., 2020). Online classes provided a safe and secure means of engaging with students to continue learning, hence, almost all higher education institutions globally, shifted to online-classroom learning. This huge unanticipated transition from the traditional on-sight learning approach to an exclusively online learning setup presented a new phase of teaching methods across educational institutions in delivering the course content to their students (Xu and Jaggars, 2011). Unlike higher education students in developed countries, who are already exposed to online textbooks and modules with video lectures and computer-based exams in the 21st century, many students in low-economy countries like Kenya are new to online-classroom learning. Online learning can be challenging for students who are being exposed to it for the first time, and also because of the limited non-verbal communication. Other aspects of the online classroom, such as student and instructor interactions or engagement, accessibility of learning materials, internet access and cost, perceived quality, self-confidence, and time management, can equally influence the overall experience of online education participants and their satisfaction (Appleton-Knapp and Krentler, 2006; Kuo et al., 2014; She et al., 2021; Conrad et al., 2022). It is therefore important to assess this evolution in teaching modalities, to provide policymakers with data meant to improve the teaching and learning process in these odd periods.

Regardless of the fact that many students in developing countries such as Kenya are only experiencing the exclusive online learning approach for the first time, research on students’ experience and satisfaction is largely lacking. It is expected that the abrupt introduction of the online classroom would present several challenges that bother on issues such as internet accessibility and affordability, perceived quality of the learning process and activities, teacher-student engagement, as well as overall students satisfaction, all of which influence the success of this learning method. Therefore, there is a need to assess the effect of students’ online-learning experience on their satisfaction in Kenya during the COVID-19 pandemic. Moreover, since the COVID-19 outbreak resulted in a sudden dramatic change that left no time and space for preparing students to adopt and accept online learning (as against traditional on-sight classroom learning), students’ preference for online learning could significantly influence their overall satisfaction with online-learning sections. Thus, examining the direct and mediating effect of students’ preference on their online-learning satisfaction could emphasize the need to properly and adequately orient students to accept online learning as an alternative to the traditional on-sight learning approach. This study assesses the effect of the online learning experience of university students on their satisfaction in the setting of the COVID-19 pandemic in Kenya. The mediating role of students’ preference on the relationship between online-learning experience and students’ satisfaction is also examined. This is expected to provide means of improving e-learning and maximizing students’ satisfaction in the phase of both pandemic and normal times.

## 2. Literature review and hypotheses development

Online learning is a global trend in higher education, particularly in the context of the corona virus disease (COVID-19) pandemic. Scholars from different countries and institutions are eagerly exploring innovative online teaching and learning strategies intending to enhance student achievement in terms of perceived learning satisfaction and engagement (Muzammil et al., 2020; Yousaf et al., 2022). Previous studies have suggested that heightened learning satisfaction may lead to further engagement, which is a significant predictor of learning outcomes (Yousaf et al., 2022). Students’ satisfaction is influenced by varying factors from two main sources, which are personal and institutional factors (Marzo Navarro et al., 2005; Appleton-Knapp and Krentler, 2006). The personal factors cover individual aspects such as age, gender, preferred learning method, and student GPA, while the institutional factors cover the quality of instructions, the quality of the classroom, course content and learning materials, promptness and quality of the instructor’s feedback, teaching style, available learning equipment, and clarity of expectation. Moreover, the effective use of technology, the quality of lecturers, and the quality of physical facilities as key determinants of student satisfaction (Wilkins and Stephens Balakrishnan, 2013). Other factors such as lecturer-student relationship, interaction with fellow students, teaching ability, and flexible curriculum influence learners’ preference and satisfaction (Fisher et al., 2021; Kim and Kim, 2021). Previous studies on e-Learning, including that of Unver et al. (2017), Szopiński and Bachnik (2021), and Milicevic et al. (2021) used different aspects of students’ online-learning experience such as teaching performance and engagement, acquisition of self-confidence, internet access and cost and perceived quality to assess the overall students’ satisfaction with online-learning (Unver et al., 2017; Milicevic et al., 2021; Szopiński and Bachnik, 2021). Other studies have also examined the relationship between students’ preference for online learning and their satisfaction during the COVID-19 pandemic (Segbenya et al., 2022). Several studies have proposed theories that explain the relationship between student experience and satisfaction in an effort to better understand the psycho-social dynamics of student satisfaction, including the expectation confirmation theory (Oliver, 1977, 1980), happy-productive” student theory (Cotton et al., 2002), and investment model theory (Hatcher et al., 1992). This study employed the expectation confirmation theory to explore the relationship between students’ online learning experience and their satisfaction. As a theoretical approach based on consumer satisfaction, the expectation confirmation theory considers satisfaction as a function of the extent to which students’ expectations about online learning are met, with positive confirmations resulting in higher levels of satisfaction (Jiang and Klein, 2009).

### 2.1. The effect of teaching performance and engagement on students’ satisfaction

Tertiary institutions remain committed to enhancing students’ learning outcomes, which have always been evaluated in terms of student engagement, performance, and satisfaction (Fisher et al., 2021). Student engagement is characterized by the degrees of attention, interest, participation, curiosity, optimism, belonging, passion, in-depth learning, interaction, and a sense of autonomy and control experienced by students (Deschaine and Whale, 2017; Fisher et al., 2021). Engagement involves more than participation in an activity; it also includes feelings, emotions, and finding value in an experience. Therefore, student engagement involves expending effort and time on learning (Alaulamie, 2014). Learning satisfaction which positively correlates with learning engagement is identified as a key indicator of a student’s enjoyment of their studies, where engagement serves as an essential construct for academic success (Bond et al., 2021; Fisher et al., 2021). Thus, sufficient teaching engagement leads to increased students satisfaction (Kim and Kim, 2021; She et al., 2021; Yousaf et al., 2022). It is further demonstrated that the instructor’s ability to deliver quality E-learning (quality teaching performance) affects students’ satisfaction (Pham et al., 2019). Quality teaching includes sufficient teacher-student engagement. Studies show the importance of quality learner-instructor interaction as two-way communication between the instructor and students (Alqurashi, 2019), and is linked with teaching performance. Besides, it is documented that learner-content engagement or interaction is the most important predictor of student satisfaction (Kuo et al., 2014; Alqurashi, 2019). Thus, teaching performance and engagement significantly influence students’ satisfaction and serve as critical markers of effective teaching and students’ satisfaction (Carpenter et al., 2020) and vice versa (Yılmaz and Yılmaz, 2022). From the perspective of the expectation confirmation theory, students expect better teaching performance and engagement in their online classes, and the degree to which demand is met influences heir satisfaction (Jiang and Klein, 2009).

### 2.2. The effect of internet accessibility and cost on student satisfaction

Students use the internet daily to access information, gather data, and conduct research. In the phase of the COVID-19 pandemic, internet usage became the only option for most educational facilities owing to the lockdown of entire regions and cities (Imsa-ard, 2020; Bond et al., 2021). Despite the wide adoption of online learning in higher education during the COVID-19 pandemic, several factors that negatively influence students’ satisfaction with this novel learning environment, such as internet accessibility and affordability, still remain in many countries, as studies indicate differences in student access to digital learning resources while at home, including high-quality broadband connectivity (Rasheed et al., 2020; Cullinan et al., 2021). Students who experience internet connectivity problems such as network congestion during online learning are found to poorly rate their e-Learning experience and their overall satisfaction (Li et al., 2021). Internet cost and accessibility remain a challenge globally, even in developed countries. For example, a study in the US estimated that 20% of college students had difficulty maintaining access to technology due to internet connectivity problems and data limitations (affordability) (Gonzales et al., 2020). The challenge of internet affordability and accessibility is driven by a range of factors, including financial constraints, gaps in access to appropriate equipment such as a laptop or desktop personal computer, and the digital literacy skills required to engage with online learning (Silva et al., 2018). Variations in connectivity constrain student engagement in online class and with online content, invariably affecting students’ performance and satisfaction (Gonzales et al., 2020). On the background of the expectation confirmation theory, students anticipate smooth internet connectivity that is also cost-effective to have a posiive learning experience, which inturn increases satisfaction (Oliver, 1980; Jiang and Klein, 2009).

### 2.3. The relationship between perceived quality and student satisfaction

In addition to students’ preference and teaching performance and engagement, perceived quality (perception of online learning being well delivered), is reported to be highly important in determining the students’ satisfaction (Ho et al., 2021). Students are more satisfied with online learning if they generally perceive an online course as quality, appropriate, and like the online course, or somewhat familiar with the course background (Beqiri et al., 2009). A study conducted during the COVID-19 pandemic lockdown in Thailand found that, regardless of the abrupt move from traditional classrooms to online learning, students’ expectancy of the quality (perceived quality) of the newly introduced learning system was matched with the traditional face-to-face learning and influenced their satisfaction. Therefore, the perceived quality of the online-learning system forms a significant part of overall student satisfaction (Kornpitack and Sawmong, 2022). Interestingly, students’ perception of quality teaching remains an essential part of their learning experiences in school and later in life (Muvui Muya, 2019). Therefore, students’ satisfying experience with traditional on-sight learning might cause them to highly expect quality teaching and learning experiences from the online-learning platforms, thus contributing to their overall satisfaction. Drawing on the expectation confirmation theory, perceived quality as an expectation construct, will influence perceived performance and attract either a positive or negative evaluation (disconfirmation of beliefs), invariably affecting satisfaction (Oliver, 1977, 1980).

### 2.4. Self-confidence and student satisfaction

Students’ confidence in online leaning was reported as the strongest positive predictor of both students’ satisfaction and perceived quality or usefulness of online classes (Landrum, 2020). Self-confidence is defined as one’s belief in his/her ability to perform best, capacity to maximize self-faith, and believing in self-worth, and serves as a crucial determinant of academic performance (Ballane, 2019). Students with high self-confidence turn to welcome new challenges and have a greater desire to learn. It is reported that students need not only the knowledge of the subject to reach their learning objectives in e-learning but also self-confidence (Kaleci and Akleman, 2019). Since the pandemic-imposed changes affect the psychological well-being of students (Villani et al., 2021), where online learning poses threats to self-confidence as it could instill fear, disappointment, and shame (Blanco et al., 2020), the acquisition of self-confidence would influence students satisfaction. Self-efficacy, which also reflects self-confidence in online learning, refers to one’s confidence to use the necessary gadget and the internet to search for information (Landrum, 2020), and positively correlates with students’ online learning satisfaction (Kirmizi, 2015; Yilmaz, 2017; Hammouri and Abu-Shanab, 2018) as well as their perceived ease of use, quality, and usefulness (Chen et al., 2020). Other studies on the COVID-19 outbreak report the direct and indirect influence of self-efficacy and the perceived ease of use and usefulness of online platforms on students’ satisfaction (Jiang et al., 2021). Thus, students’ e-learning self-confidence and readiness are significant predictors of their satisfaction and motivation (Yilmaz, 2017). Drawing on the expectation confirmation theory, acquisition of self-confidence will lead to a positive disconfirmation, which is posited to increase post-online learning or post-adoption satisfaction (Oliver, 1977, 1980; Jiang and Klein, 2009).

### 2.5. Effect of students’ online learning experience on preference

Student online learning experience, including poor internet access and connectivity, discomfort, and lack of familiarity with the technology, negatively influence students’ preference for online learning (DeLone and McLean, 2003; Al-Fraihat et al., 2020). A survey carried out in 2020 that focused on technological issues and challenges during the transition to online learning, found that the lack of readiness coupled with internet access issues was directly associated with the online-learning system quality, and significantly influenced student satisfaction (EDUCAUSE, 2020), as assumed in the original model of Delone and Mclean (DeLone and McLean, 2003; Al-Fraihat et al., 2020). Similarly, other literature suggests that improved system quality positively influences student preference and satisfaction when E-learning (Cidral et al., 2018; Al-Fraihat et al., 2020). Self-efficacy, which also reflects self-confidence in one’s ability, is defined as the individuals’ belief in their own capability to perform a certain task, challenge, or successfully engage with educational technology influences students’ readiness and preference for online-learning technologies (Eom, 2012; Patricia Aguilera-Hermida, 2020), and has been shown to be interconnected with student satisfaction levels (Wang and Degol, 2014). Self-efficacy is affected by online platform content and accessibility, which in turn, positively influence student satisfaction (Prifti, 2022). Moreover, in the application of technology in teaching and learning, adequate orientation and training of students and faculty in remote learning and teaching may enhance preference (Muthuprasad et al., 2021), as indicated in recent reports of increased students’ preference for online leaning (Jenay, 2022). The success in employing e-learning is also associated with quality teaching performance, an interactive teaching style, and attitudes of the teacher, as well as the attitudes and experiences of students with respect to technology (Linjawi, 2010), all of which are influenced by preference. From the perspective of the expectation confirmation theory, if students online learning expectations are met, it will positively influence their preference for the online learning classroom and vice versa (Jiang and Klein, 2009).

### 2.6. The relationship between student preference and satisfaction

It is reported that students’ preference for either online learning or on-sight learning significantly influences their overall satisfaction with learning. For example, a study found that although the majority of students were competent in technology and had no obvious challenge in accessing learning devices or Wi-Fi during the COVID-19 pandemic, they simply preferred face-to-face learning to online learning, and this preference was found to be the most important predictor of students’ satisfaction (Ho et al., 2021). Since most students were only engaged in the traditional face-face teaching and learning process before the pandemic, the lack of adequate orientation and ample time to adjust to the online-learning process leads to less preference and lack of satisfaction (Karadag et al., 2021). This is also asserted by other researchers who indicate that typically online learning is regarded as a well-planned system from the beginning and may go through a lengthy designed process (Charles et al., 2020), however, the online teaching and learning systems being employed in many countries were hurried to provide a shift in instructional delivery due to the COVID-19 crisis (Cameron-Standerford et al., 2020; Rahiem, 2020). Therefore, decreased preference or readiness negatively influences satisfaction (Rahiem, 2020). Drawing on the expectation confirmation theory, increased preference will correlate with a positive evaluation or disconfirmation of beliefs, and will lead to increased satisfaction (Oliver, 1977, 1980).

In summary, students have been forced into online classrooms due to the COVID-19 pandemic. On the phase of the implication, several factors can undermine the success of online learning, thus it is important to assess and understand the perspective of the student regarding their experience with online learning during the pandemic, and how it influences their overall satisfaction with online learning.

### 2.7. Conceptual framework

**Figure 1:**
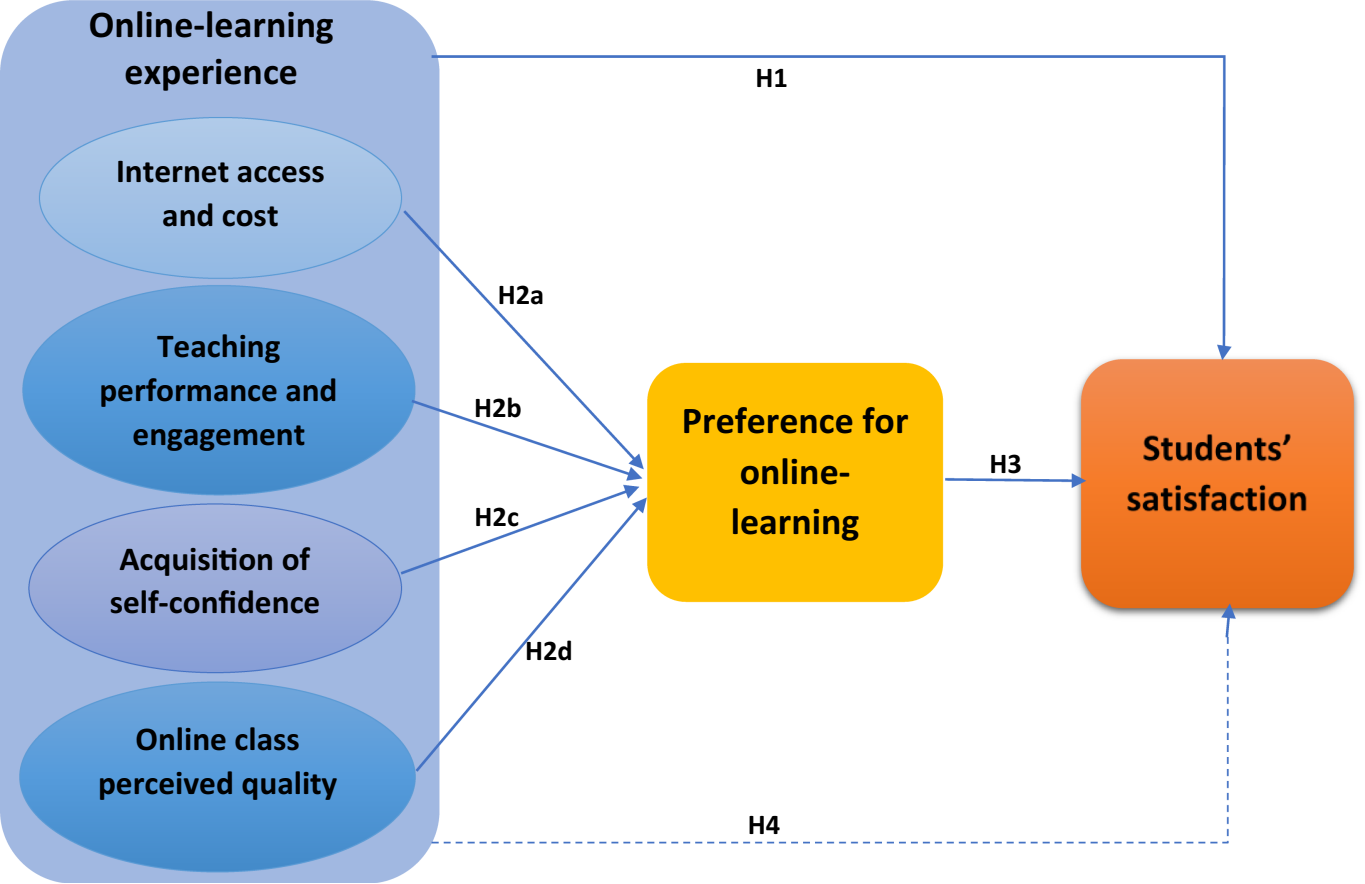
The conceptual framework of the study.

The theoretical foundation of our framework is based on the Expectation Confirmation Theory by Richard Oliver, which is a cognitive theory that seeks to explain post-adoption satisfaction as a function of users’ expectations, perceived performance, and disconfirmation of beliefs. Thus, the primary construct of this theory are expectations, perceived performance, disconfirmation of beliefs, and satisfaction (Oliver, 1977, 1980). Expectations refer to users’ anticipated or predicted attributes associated with the service or technology artifact and directly affect both perceived performance (users’ perceptions of the actual performance of a service or technology artifact) and disconfirmation of beliefs (service or technology artifact evaluation or judgment) and indirectly affect post-adoption satisfaction by way of a mediational relationship through the disconfirmation construct (Oliver, 1977, 1980; Bhattacherjee, 2001). In the light of the Expectation Confirmation Theory, users’ expectations and perceived performance of the online learning platform constitute the students’ online learning experience (perceived quality of online classroom learning, teaching performance and engagement, internet access and cost, and acquisition of self-confidence), which influence their disconfirmation (preference). The disconfirmation of beliefs, herein represented by preference, as an evaluation of the online learning service produces either a positive or negative response, which in turn influences users’ satisfaction (Bhattacherjee, 2001). In addition, users experience with the online learning directly influences their satisfaction (Figure 1).

Moreover, several factors have been reported that identify and influence students’ online learning satisfaction (Al-Fraihat et al., 2020). An earlier online-learning study model proposed by DeLone and McLean (2003), primarily considered factors such as the quality of information systems and services that determine learner satisfaction (DeLone and McLean, 2003). This model has been used to assess online-learning success among students in universities during the COVID-19 pandemic (Shahzad et al., 2021). Moreover, the user satisfaction approach (Al-Fraihat et al., 2020), as a theoretical framework in the assessment of E-learning, has been adopted by researchers to measure the learners’ satisfaction during their online learning experience in higher education in developing countries (Yawson and Yamoah, 2020). Other E-learning quality and technology acceptance models have been developed with an emphasis on user experience that culminates into the platform’s usefulness and ease of use (Abdullah and Ward, 2016; Al-Fraihat et al., 2020). Conceptual models based on a semi-structured questionnaire conducting thematic analyses of college students’ online learning experience during the COVID-19 pandemic (Shim and Lee, 2020), as well as tailor-made questionnaires that measure student satisfaction using 5-point Likert-scale questions (Alqurshi, 2020) have been developed.

However, most of the available theoretical models are built to assess pre-planned E-learning but not the abruptly imposed online learning seen during the COVID-19 pandemic, thus, their direct application may not suitably reflect underlying factors influencing the satisfaction and success of the COVID-19-induced emergency remote learning. Therefore, some researchers have developed other frameworks, such as a tailor-made survey kit by EDUCAUSE that allows institutions to rapidly adopt to gather feedback from higher education stakeholders (EDUCAUSE, 2020). This gives room for framework formation that is carefully tailed to adequately assess the online-learning situation in Kenya while taking reference from the components of the multidimensional EESS (Evaluating E-learning System Success) model. Therefore, in this study, students’ online study experience was assessed by considering some of the key factors that influence effective e-Learning, including internet access and cost, teaching performance and engagement, perceived quality of online classroom learning, and acquisition of self-confidence (Unver et al., 2017; Milicevic et al., 2021; Szopiński and Bachnik, 2021). In the light of the multidimensional EESS model, teaching performance and engagement falls under instructor quality, students’ self-confidence and preference under learner quality, internet access and cost under information quality – accessibility, and perceived quality of online learning under educational system quality (Al-Fraihat et al., 2020). These factors together shape a student’s learning experience and lead to the overall satisfaction of the entire learning process. Thus, the effect of students’ online study experience on their satisfaction was examined. Moreover, the influence of students’ preferences on their satisfaction was assessed. Finally, the mediating role of students’ preference on the relationship between their online-learning experience and satisfaction was examined (Figure 1).

### 2.8. Hypothesis

From the background reviewed in the literature and the conceptual framework (Figure 1), four main hypotheses were deduced for the study.

*HI*: There is a direct significant relationship between students’ online learning experience and their overall satisfaction with online classes during the COVID-19 pandemic.

*H1a*: Internet access and cost influence students’ overall satisfaction with online classes during the COVID-19 pandemic.

*H1b*: Teaching performance and engagement have a significant positive influence on students’ overall satisfaction with online classes during the COVID-19 pandemic.

*H1c*: Students’ acquisition of self-confidence through online learning during the COVID-19 pandemic has a significant positive influence on students’ overall satisfaction.

*H1d*: Students’ perceived quality of online learning has a significant positive influence on students’ overall satisfaction during the COVID-19 pandemic.

*H2*: Students’ online-learning experience significantly correlates with their preference for online learning during the COVID-19 pandemic.

*H2a*: Internet access and cost influence students’ preference for online learning during the COVID-19 pandemic.

*H2b*: Teaching performance and engagement have a significant positive influence on students’ preference for online learning during the COVID-19 pandemic.

*H2c*: Students’ acquisition of self-confidence through online learning during the COVID-19 pandemic has a significant positive influence on students’ preference for online learning.

*H2d*: Students’ perceived quality of online learning has a significant positive influence on students’ preference for online learning during the COVID-19 pandemic.

*H3*: Students’ preference for online learning has a significant positive influence on students’ overall satisfaction during the COVID-19 pandemic.

*H4*: Students’ preference for online learning mediates the effect of students' online learning experience on their overall satisfaction during the COVID-19 pandemic.

*H4a*: Students’ preference for online learning mediates the effect of internet access and cost on their overall satisfaction with online classes during the COVID-19 pandemic.

*H4b*: Students’ preference for online learning mediates the effect of teaching performance and engagement on their overall satisfaction with online classes during the COVID-19 pandemic.

*H4c*: Students’ preference for online learning mediates the effect of students’ acquisition of self-confidence through online learning on their overall satisfaction during the COVID-19 pandemic.

*H4d*: Students’ preference for online learning mediates the effect of students’ perceived quality of online learning on their overall satisfaction during the COVID-19 pandemic.

## 3. Materials and methods

### 3.1. Research design and study area

A cross-sectional study design was used to obtain the primary data from university students between January 2021 and June 2021. Participants were selected from universities in Nairobi, also known as the safari capital of Africa, and serves as the capital and largest city of Kenya.

### 3.2. Sampling technique

The study employed the convenient simple random sampling approach as it is considered reliable, fair, and effective. A survey form was prepared by using the Microsoft Form web-based survey technology and that access link was distributed among students in the selected universities. Participants received the survey link through social networks such as WhatsApp and Instagram, which contained clearly outlined questions and instructions. Respondents could take part and complete the questionnaire at any time of their convenience.

### 3.3. Sample population and size

Participants were recruited among students from three selected universities in Nairobi, i.e., the Kenyatta University, The Technical University of Kenya, and the Jomo Kenyatta University of Agriculture and Technology. A total of 501 respondents made up of males and females who were willing to participate were recruited. The criteria for selection included only students who have experienced or currently experiencing online-classroom learning owing to the COVID-19 pandemic.

### 3.4. Instruments for data collection

Validated questionnaires used in previous studies on the subject in different parts of the world including the studies by Unver et al. (2017), Szopiński and Bachnik (2021), Pham et al. (2019), Segbenya et al. (2022), and Fieger (2012) were used. The questionnaires involved a Likert scale of 1–5 to assess the various aspects of students’ experience in the online-learning classroom (i.e., internet access and cost, teaching performance and engagement, online classroom perceived quality, and acquisition of self-confidence in the online classroom) and their effect on students’ satisfaction, where ‘5’ was an opinion indicating that the student strongly agreed and ‘1’ was an indicator that the student strongly disagreed. Details of the variables and their items used for the study can be found in the Supplementary material. The questionnaire was made up of two sections; background information of respondent (sex, age, education level, frequency of participation in online leaning, and preference) and assessment of online learning. The assessment of frequency of participation in online leaning and preference for online or offline leaning was deducted from the work of Szopiński and Bachnik, (2021). The two items for assessing internet access and cost (IAC1 and IAC2) were deducted from the questionnaire used in the study by Segbenya et al. (2022), while the four items used to measure online class perceived-quality (OCPQ1 – OCPQ4) were extracted from Pham et al. (2019). The questionnaire on teaching performance and engagement was deducted from the study by Unver et al. (2017) (TPE2 and TPE3) and Fieger (2012) (TPE1 and TPE4), whereas all four items used to measure self-confidence (ASC1 – ASC4) were deducted from the study of Unver et al. (2017). The questions on preference for online learning (POL1 – POL4) were deducted from the work of Segbenya et al. (2022) and overall students satisfaction (SS) from Alqurashi, (2019) and Fieger, (2012). The internal consistency of the questionnaire was checked and the CFA loadings of all variables had a significant value of p of <0.001 and reliability Cronbach’s alpha coefficient greater than the recommended threshold of 0.7 (except for internet access and cost-IAC, *α* = 0.643). Since an AVE < 0.50 but >40 with an α value <0.6 is acceptable, all the variables in this study were valid and reliable for the dataset. In other words, the results indicated that the scales had satisfactory internal consistency and acceptable convergent validity.

### 3.5. Instrument validity/reliability

The questionnaire was subjected to review by the researcher’s colleagues and a pilot test among a few participants to ensure that any irrelevant material, contradictions, spelling errors, offensive language, and discrepancies were eliminated. This also ensured that ambiguity was eliminated and that sensitive questions were rephrased or avoided entirely. Moreover, the test of the reliability of the questionnaire by means of Cronbach’s coefficient of reliability indicated internal consistency (Table 1).

**Table 1:** Results of CFA loadings, reliability, and validity.

	Standardized *β*	S.E.	C.R.	*p*	*α*	Composite reliability	AVE
OCPQ4 < --OCPQ	0.916				0.926	0.927	0.760
OCPQ3 < --OCPQ	0.897	0.032	30.938	***			
OCPQ1 < --OCPQ	0.859	0.033	28.096	***			
OCPQ2 < --OCPQ	0.81	0.036	24.885	***			
ASC4 < --ASC	0.815				0.899	0.901	0.694
ASC3 < --ASC	0.893	0.045	23.344	***			
ASC2 < --ASC	0.82	0.047	20.901	***			
ASC1 < --ASC	0.801	0.051	20.236	***			
TPE3 < --TPE	0.837				0.885	0.886	0.661
TPE2 < --TPE	0.849	0.047	22.138	***			
TPE1 < --TPE	0.776	0.049	19.585	***			
TPE4 < --TPE	0.787	0.045	19.964	***			
POL2 < --POL	0.987				0.847	0.88	0.655
POL3 < --POL	0.813	0.029	26.984	***			
POL1 < --POL	0.594	0.054	15.681	***			
POL4 < --POL	0.794	0.028	25.64	***			
IAC1 < --ICC	0.724				0.643	0.645	0.477
IAC2 < --ICC	0.656	0.211	4.451	***			

S.E., standard error; C.R., critical ratio; *α*, Cronbach’s alpha; AVE, average variance extracted. ***p < 0.001. Abbreviations: OCPQ, online classroom perceived quality; ASC, acquisition of self-confidence; TPE, teaching performance and engagement; IAC, internet access and cost.

### 3.6. Data collection and analysis

The application of Google Docs in designing the online questionnaires made the data collation simpler since the total data collected was summarized and presented on a spreadsheet. The data was analyzed using the IBM® SPSS® software platform (version 26) and AMOS (version 26) software, where both descriptive and inferential statistics were conducted. Frequencies, means, and standard deviations were applied to describe the demographics of participants and examine their experience, preference, and satisfaction concerning online learning. Moreover, correlation analysis was applied to determine whether or not there was a significant relationship between the research variables, by comparing the means. In addition, a structural equation model (SEM) was used to analyze the students’ responses to examine the effect of students’ online-learning experience on their satisfaction, as well as the direct and mediation influence of students’ preferences on this relationship. This simulation was carried out by measuring, assessing, and calculating the constraints or the parameters, including exploratory factor analysis (EFA) with SPSS® and confirmatory factor analysis (CFA) with AMOS. In this process, the validity, reliability, and construct loadings were performed. The path coefficient, predictive accuracy (R2), effect size (f2), and predictive relevance (Q2) were also calculated. All statistically significant values were set at a significance level of *p* ≤ 0.05.

#### 3.6.1. Reliability and validity of measurement model

Validity describes the extent to which a measurement item truly measures what it is expected to measure, while reliability describes an instrument’s consistency (Diamantopoulos and Temme, 2013; Lowry and Gaskin, 2014; Gaskin and Lim, 2016). Concerning the validity of the indicators, the researcher examined the paths’ weight and significance, linking each latent variable to its observed variables. The observed variables’ loadings should be significant (*p* < 0.05 or better), and the t-values are expected to be 1.96 in absolute terms. The reliabilities of the observed variables were assessed by examining the squared multiple correlations. A higher multiple-squared correlation value signifies the observed variable’s high reliability (Boduszek et al., 2013).

#### 3.6.2. Assessment of model fit

These indices employed in the model fit assessment included Chi-square (x^2^), the normed fit index (NFI), the standardized root mean square residual (SRMR), the comparative fit index (CFI), the root mean error square of approximation (RMSEA), and the goodness of fit index (CFI) as earlier indicated (Gaskin and Lim, 2016).

#### 3.6.3. Structural equation model

The structural equation model (SEM) was employed since it allows simultaneous evaluation of model construct relationships. The SEM served as not just a predictive model with a column vector, y, containing p-dependent variables, but also explicitly formulated as a causal model.

#### 3.6.4. Assessment of the structural path model

To evaluate the structural aspect of the model, the paths linking the different independent variables (students’ online-learning experience consisting of internet access and cost, teaching performance and engagement, perceived quality of online-classroom learning, and acquisition of self-confidence), mediating variable (students’ preference) and the dependent unobserved variable (students’ satisfaction) were examined to determine whether the hypothesized relationships (H1, H2, H3, and H4) were supported by the data. The parameter signs linking the unobserved variables were also examined to establish adequate support for the hypothesized relationships. Moreover, the weight and significance of the parameter estimate and the squared multiple correlations (*R*^2^) were estimated to know the level of variance.

## 4. Results

### 4.1. Sociodemographic of respondents

Out of the 501 respondents, 296 (59.1%) were males. Approximately half of the participants (253/501, 50.5%) were between 18 and 28 years and 215 (42.9%) were between 29 and 39 years. The participants were well-distributed between the different levels of university education to prevent skewed data, as approximately 31% represented undergraduate, 46% master, and 23% Ph.D. students. Other details on the sociodemographic are presented in Table 2.

**Table 2:** Sociodemographic of respondents.

Sociodemographic		Frequency	Percent	Valid percent	Cumulative percent
Gender	Male	296	59.1	59.1	59.1
	Female	205	40.9	40.9	100.0
	Total	501	100.0	100.0	
Age	18–28 years	253	50.5	50.5	50.5
	29–39 years	215	42.9	42.9	93.4
	40–50 years	33	6.6	6.6	100.0
	Total	501	100.0	100.0	
Education	Undergraduate	155	30.9	30.9	30.9
	Master	231	46.1	46.1	77.0
	PhD	115	23.0	23.0	100.0
	Total	501	100.0	100.0	

### 4.2. Frequency of participation and preference for online classes

All respondents had participated in online classes before and during the COVID-19 pandemic. While approximately 32% were likely to have an increase in online class participation, 30% rather anticipated a decrease in online classroom learning. Interestingly, about 39% of the respondents indicated that their participation frequency in online classes would likely not change (Table 3). There is a profound variation of the online learning environment from the traditional in-person classroom situation regarding outcomes such as learner satisfaction, motivation, and interaction (Bignoux and Sund, 2018). On the phase value of their experience in the online classes, approximately 80% (402/501 participants) indicated their preference for in-person learning as against online learning (Table 3).

**Table 3:** Frequency of participation and preference for online classes.

	Frequency	Percent	Valid percent	Cumulative percent
Frequency				
I’m more likely to participate in online classes than before	158	31.5	31.5	31.5
I’m less likely to participate in online classes than before	148	29.5	29.5	61.1
My participation frequency has not changed	195	38.9	38.9	100.0
I have never participated in an online class	000	000	000	100.0
Total	501	100.0	100.0	100.0
Preferences				
I prefer in-person (offline) classes most	402	80.2	80.2	80.2
I prefer online classes most	99	19.8	19.8	100.0
Total	501	100.0	100.0	

### 4.3. Students’ opinions on online learning

The questionnaire employed a five-level scale with ‘5’ as an opinion indicating that the student strongly agreed and ‘1’ as an indicator that the student strongly disagreed. Interpretation and criteria values were 4.50–5.00 indicating ‘strongly agreed’, 3.50–4.49 indicating ‘agreed’, 2.50–3.49 indicating ‘neutral’, 1.50–2.49 indicating ‘disagreed’, and 1.00–1.49 indicating ‘strongly disagreed’ (Ruenphongphun et al., 2021) to assess the students’ opinion concerning their online learning experience. The variable with the lowest score was teaching performance and engagement-TPE (*M* = 2.59, SD = 0.97), followed by the acquisition of self-confidence-ASC (*M* = 2.70, SD = 0.99) and online classroom perceived quality-OCPQ (*M* = 2.89, SD = 1.06) as presented in Table 4. The neutral mean response indicates that although the students do not agree that they had a good online learning experience, they also disagree that it was poor. Neutral mean response scores were also recorded for internet access and cost-IAC (*M* = 3.04, SD = 0.82), preference for online learning-POL (*M* = 3.24, SD = 0.90), and students’ satisfaction-SS (*M* = 3.26, SD = 1.17). The high standard deviations noticed indicate that the data are more spread out; more variable in students’ opinions concerning their online-learning experience and satisfaction (McGrath et al., 2020). However, only about 20% (99/501) of the students indicated their preference for online-learning relative to 80% who preferred face-to-face learning (Table 3). The abrupt introduction of online classes without prior orientation and training might have contributed to the low preference or acceptance rate among the students.

**Table 4:** Correlation analysis.

	Gender	Age	Education	Frequency	Preferences	SS	IAC	OCPQ	TPE	POL	ASC
Gender	1										
Age	−0.099*	1									
Education	0.058	0.380**	1								
Frequency	−0.239**	0.234**	0.059	1							
Preferences	0.127**	0.151**	0.130**	−0.194**	1						
SS	−0.161**	0.117**	0.269**	0.201**	0.337**	1					
IAC	−0.133**	0.178**	−0.420**	0.057	−0.093*	−0.275**	1				
OCPQ	−0.057	0.351**	0.245**	0.126**	0.288**	0.267**	−0.026	1			
TPE	−0.212**	0.146**	0.243**	0.051	0.179**	0.407**	−0.034	0.184**	1		
POL	−0.300**	−0.018	0.153**	0.217**	0.065	0.772**	−0.200**	0.208**	0.502**	1	
ASC	−0.380**	0.391**	0.280**	0.262**	0.141**	0.434**	0.021	0.303**	0.558**	0.523**	1
Mean	1.41	1.56	1.92	2.07	1.20	3.26	3.04	2.89	2.59	3.24	2.70
Std. Deviation	0.49	0.62	0.73	0.84	0.40	1.17	0.82	1.06	0.97	0.90	0.99

*p < 0.05; **p < 0.01. OCPQ, online classroom perceived quality; ASC, acquisition of self-confidence; TPE, teaching performance and engagement; POL, preference for online learning; IAC, internet access and cost; SS, students’ satisfaction.

### 4.4. The relationship between students’ online-learning experience (IAC, OCPQ, TPE, ASC), preference for online learning (POL), and students’ satisfaction (SS)

To examine the relationship between students’ online-learning experience and their overall satisfaction (SS) with online classes during the pandemic, correlation analysis was carried out (Table 4) using Pearson Moment Correlation (r). Students’ online-learning experience was assessed using their responses to OCPQ, ASC, TPE, IAC, and POL. Results showed that SS had a significant positive correlation with OCPQ (*r* = 0.267, *p* = 0.01), ASC (*r* = 0.434, *p* = 0.01), TPE (*r* = 0.407, *p* = 0.01), and POL (*r* = 0.772, *p* = 0.01) but a negative correlation with IAC (*r* = −0.275, *p* = 0.01). Moreover, while POL positively correlated with OCPQ, ASC, and TPE, there was rather a negative correlation of POL with IAC (Table 4). This means that students develop a better experience with the online classes once there is increased online class perceived quality, enhanced teaching performance and engagement, and acquisition of self-confidence, leading to overall satisfaction. On the other hand, internet access and cost negatively influence student satisfaction.

### 4.5. Exploratory factor analysis

SPSS was employed to perform exploratory factor analysis (EFA). The rotated component matrix results obtained from the EFA were examined to know how the measures of the various parameters being considered in the study (i.e., OCPQ, ASC, TPE, POL, and IAC) were loaded onto their suggested constructs. The Kaiser-Meyer-Olkin Measure (KMO) of Sampling Adequacy and Bartlett’s Test of Sphericity were checked to ascertain whether the samples were sufficient to carry out the survey analysis. The amount of variance explained by the factors was also measured. The study analyzed the overall job satisfaction construct with one item, hence, it was not included in the EFA.

#### 4.5.1. Kaiser–Meyer–Olkin Bartlett’s test

The EFA analysis produced a Kaiser-Meyer-Olkin Measure of Sampling Adequacy value of 0.869 with a value of p of less than 0.001 (Table 5). This indicates that the sample was sufficiently adequate for the study.

**Table 5:** Determination of sample sufficiency.

KMO and Bartlett’s test		
Kaiser–Meyer–Olkin measure of sampling adequacy		0.869
Bartlett’s test of sphericity	Approx. Chi-Square	6156.183
	df	153
	Sig.	0.000

#### 4.5.2. Eigenvalues and variances of the study variables

Eigenvalues express the total variance that could be explained by a given principal component. The Eigenvalues again. Represent the sum of squared component loadings across every item for each component, which stands for the amount of variance in each item that can be explained by the principal component. Thus, eigenvectors represent a weight for each eigenvalue (Bruin, 2006). The EFA results showed five components with a sum eigenvalue and variance explained of 13.98 and 77.65%, respectively (Table 6).

**Table 6:** Total variance explained.

Component	Initial Eigenvalues			Extraction Sums of Squared Loadings			Rotation Sums of Squared Loadings		
	Total	% of Variance	Cumulative %	Total	% of Variance	Cumulative %	Total	% of Variance	Cumulative %
1	6.676	37.091	37.091	6.676	37.091	37.091	3.367	18.707	18.707
2	2.872	15.955	53.045	2.872	15.955	53.045	3.267	18.153	36.860
3	1.940	10.777	63.822	1.940	10.777	63.822	3.074	17.077	53.937
4	1.375	7.637	71.460	1.375	7.637	71.460	2.682	14.901	68.837
5	1.113	6.185	77.645	1.113	6.185	77.645	1.585	8.808	77.645
6	0.591	3.282	80.927						
7	0.505	2.808	83.735						
8	0.432	2.398	86.133						
9	0.351	1.947	88.080						
10	0.327	1.817	89.897						
11	0.310	1.723	91.619						
12	0.281	1.561	93.180						
13	0.264	1.465	94.645						
14	0.252	1.400	96.045						
15	0.234	1.302	97.346						
16	0.218	1.213	98.559						
17	0.169	0.941	99.501						
18	0.090	0.499	100.000						

Extraction Method: Principal Component Analysis.

#### 4.5.3. Rotated component matrix

The rotated component matrix, also known as the loadings, contains estimates of the correlations between the variables and the estimated components and serves as the main output of principal components analysis (Guilloteau et al., 2021). Further EFA analysis using the Rotated Component Matrix showed that all the factor loadings for the variables under study were greater than the suggested threshold of 0.50 (Table 7). The factor loadings ranged from 0.691 to 0.911, and they loaded well under their predicted construct. The results suggest acceptability for the items employed to measure the various constructs.

**Table 7:** EFA *via* the rotated component matrix.

Variables	Code	Component				
		1	2	3	4	5
Online classroom perceived quality (OCPQ)	OCPQ4	0.911				
	OCPQ3	0.904				
	OCPQ1	0.882				
	OCPQ2	0.868				
Acquisition of self-confidence (ASC)	ASC4		0.826			
	ASC3		0.814			
	ASC2		0.798			
	ASC1		0.797			
Teaching performance and engagement (TPE)	TPE3			0.817		
	TPE2			0.815		
	TPE1			0.801		
	TPE4			0.795		
Preference for online learning (POL)	POL2				0.869	
	POL3				0.799	
	POL1				0.722	
	POL4				0.691	
Internet access and cost (IAC)	IAC1					0.872
	IAC2					0.815

OCPQ, online classroom perceived quality; ASC, acquisition of self-confidence; TPE, teaching performance and engagement; POL, preference for online learning; IAC, internet access and cost; SS, students’ satisfaction.

### 4.6. Confirmatory factor analysis

After the EFA had identified the structure of the relationship between the variables and shown the sufficiency and validity of the dataset, the confirmatory factor analysis (CFA) was also carried out with the AMOS software to provide further reliability and validity to the data set. The CFA represents a multivariate statistical procedure employed to assess how well-measured variables act for the number of constructs and further allows the researcher to test if the hypothesis of any given relationship between an observed variable and its underlying latent construct exists (Li et al., 2020; Agegnehu et al., 2022).

#### 4.6.1. Validity and reliability of the variables in the study

In this process, the CFA loadings (*β*), which are expected to be greater than the recommended threshold of 0.50, were examined. Moreover, the data reliability was assessed using Cronbach’s alpha (*α*) and composite reliability, which are acceptable at a recommended threshold of greater than 0.70. The average variance extracted (AVE) and discriminant validity was relied on to establish the validity of the dataset. The AVE is ascribed to be better if it is greater than 0.50. However, an AVE less than 0.50 but greater than 0.40 with composite reliability greater than 0.60 can be accepted (Fornell and Larcker, 1981). The results showed AVE values greater than 0.50, except for ICC which had an AVE value of 0.477 but a composite reliability value of 0.645 (Table 1). Moreover, the CFA loadings of all variables had a significant *p*-value <0.001 and reliability Cronbach’s alpha coefficient greater than the recommended threshold of 0.7 (except for IAC, *α* = 0.643). Since an AVE < 0.50 but >40 with an *α* value <0.6 is acceptable, all the variables in this study are valid and reliable for the dataset. In other words, the results indicate that the scales had satisfactory internal consistency and acceptable convergent validity.

Discriminant validity, a subtype of construct validity was further carried out to show how well the variables measure the concept designed to measure in this study. The goal of discriminant validity evidence is to be able to discriminate between measures of dissimilar constructs (Hubley, 2014). Thus, the analysis was done to confirm that although the variables are related, they are very much distinct from each other. The results showed discriminant validity values greater than their corresponding latent variable correlation coefficients (Table 8).

**Table 8:** Correlation and discriminant validity results.

	OCPQ	ASC	TPE	POL	IAC
OCPQ	**0.872**				
ASC	0.337***	**0.833**			
TPE	0.203***	0.625***	**0.813**		
POL	0.295***	0.484***	0.521***	**0.809**	
IAC	−0.059	0.021	−0.053	−0.257***	**0.691**

***p-value < 0.001; Bold values represent discriminant. OCPQ, online classroom perceived quality; ASC, acquisition of self-confidence; TPE, teaching performance and engagement; POL, student experience on online learning; IAC, internet access and cost.

#### 4.6.2. Examining the measurement models for the various hypothesized relationships

**Figure 2:**
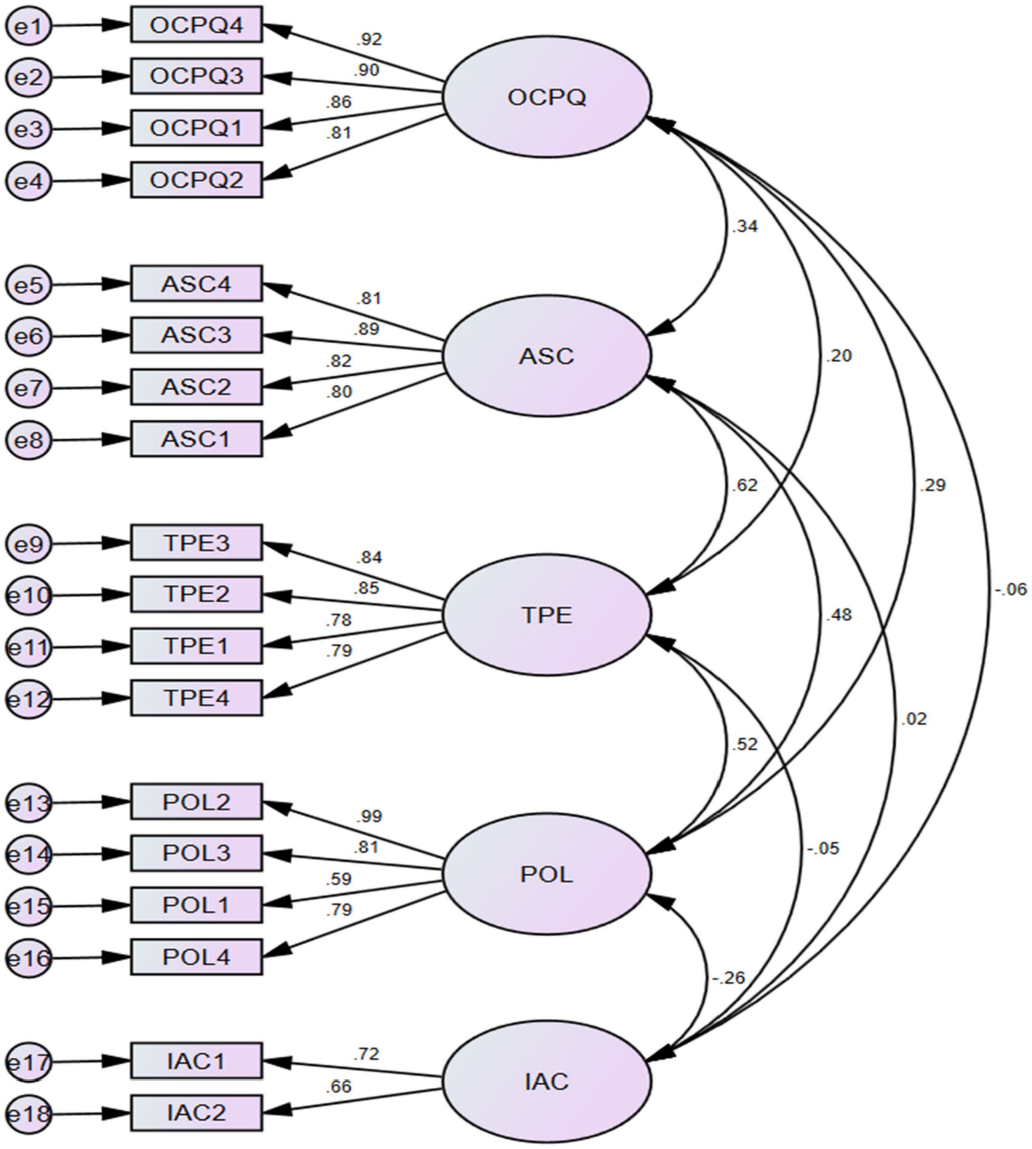
CFA Measurement Model. Model fit indices: Chi-square (CMIN) = 567.475, degree freedom (df) = 125, relative Chi-square index (CMIN/df) = 4.540, comparative fit index (CFI) = 0.927, standardized root mean square residual (SRMR) = 0.067, root mean square error of approximation (RMSEA) = 0.074. OCPQ, online classroom perceived quality; ASC, acquisition of self-confidence; TPE, teaching performance and engagement; POL, preference for online learning; IAC, internet access and cost.

The study further assessed the measurement models based on the construct’s relationships to other constructs in the model to give confidence in the structural models during hypotheses testing. Therefore, the study performed CFA with AMOS. In the process, the model fit indices such as the Chi-square (*χ*^2^), which is dependent on the sample size, standardized root mean square residual (SRMR <0.06), relative Chi-square index (*χ*^2^/df), comparative fit index (CFI > 0.95), root mean square error of approximation (RMSEA <0.06), and Bentler-Bonett normed fit-index (NFI > 0.95) were checked for acceptability. The relationship between the various parameters of students’ online-learning experience and students’ preference for online learning was examined. The students’ satisfaction variable was excluded from the CFA because it is not a latent variable. It was measured with only an item. The structure produced a model fit indices of Chi-square (CMIN) = 567.475, degree freedom (df) = 125, relative Chi-square index (CMIN/df) = 4.540, comparative fit index (CFI) = 0.927, standardized root mean square residual (SRMR) = 0.067, root mean square error of approximation (RMSEA) = 0.074. The results suggest that the study’s model fits the data set well and it is suitable for further analysis (Figure 2).

### 4.7. Hypotheses testing

The data analysis was carried out using the structural equation model (SEM) in AMOS software version 26. The SEM technique was employed to analyze the main effect involving the influence of the four facets of students’ online-learning experience (OCPQ, ASC, TPE, IAC) on students’ satisfaction with online learning. SEM was again used to analyze the mediation effect of students’ preference for online learning (POL) on their overall satisfaction as indicated in the conceptual framework. In analyzing the mediating effect of SEM, the direct paths from the students’ online-learning experience and students’ satisfaction with online learning were critically considered. The analysis was developed into the synopsis described below.

#### 4.7.1. The effect of students’ online-learning experience on students’ satisfaction

**Figure 3:**
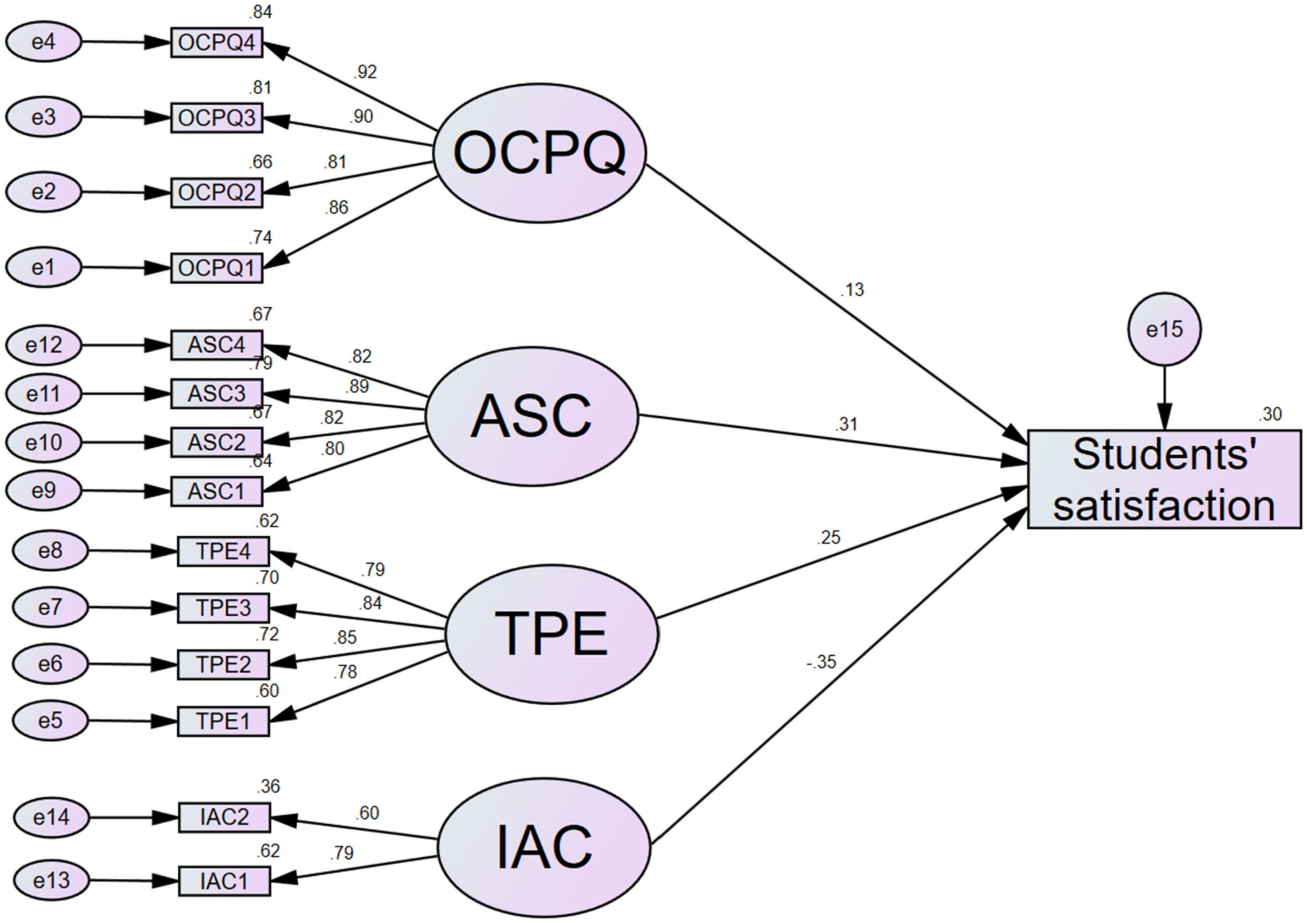
Main effect model. Model fit indices: Chi-square (CMIN) = 357.058, degree freedom (df) = 87, relative Chi-square index (CMIN/df) = 4.104, comparative fit index (CFI) = 0.940, standardized root mean square residual (SRMR) = 0.071, root mean square error of approximation (RMSEA) = 0.079. OCPQ, online classroom perceived quality; ASC, acquisition of self-confidence; TPE, teaching performance and engagement; IAC, internet access and cost.

The effects of online-learning experience (online classroom perceived quality, acquisition of self-confidence, teaching performance and engagement, internet access and cost) on students’ satisfaction was determined. Thus, the hypothesized relationship, H1 (H1a, H1b, H1c, H1d) was first tested using SEM, and the results are shown in Table 9. The structural model (Figure 3) for the main effect provided a good fit, where Chi-square (CMIN) = 357.058, degree freedom (df) = 87, relative Chi-square index (CMIN/df) = 4.104, comparative fit index (CFI) = 0.940, standardized root mean square residual (SRMR) = 0.071, and root mean square error of approximation (RMSEA) = 0.079. On the direct path without a mediator, all the four sub-hypotheses (H1a, H1b, H1c, H1d) were supported, indicating that OCPQ, ASC, and TPE had a significant positive effect on students’ satisfaction while IAC had a significant negative effect on students’ satisfaction (Table 9).

**Table 9:** The effect of students’ online-learning experience on students’ satisfaction.

Hypotheses	Path	Direct path without a mediator				Direct path with a mediator			
		Estimate	S.E.	C.R.	*p*	Estimate	S.E.	C.R.	*p*
H1a	IAC-- > SS	−0.527	0.099	−5.312	***	−0.184	0.064	−4.236	***
H1b	TPE-- > SS	0.307	0.052	5.919	***	−0.009	0.04	−0.262	0.793
H1c	ASC-- > SS	0.361	0.049	7.354	***	0.124	0.036	4.047	***
H1d	OCPQ-- > SS	0.143	0.044	3.22	0.001	0.019	0.032	0.673	0.501

***p-value < 0.001. OCPQ, online classroom perceived quality; ASC, acquisition of self-confidence; TPE, teaching performance and engagement; IAC, internet access and cost; SS, students’ satisfaction.

#### 4.7.2. Examining the structural mediation model

The three remaining components of the hypothesis were examined:

1. The effect of students’ online-learning experience on their preference for online learning, as highlighted in hypothesis H2 (H2a, H2b, H2c, H2d).

2. The effect of preference for online learning on students’ satisfaction, as highlighted in hypothesis H3.

3. Finally, the mediating roles of preference for online learning on the relationship between students’ online-learning experience and their satisfaction, as highlighted in hypothesis H4 (H4a, H4b, H4c, H4d).

**Figure 4:**
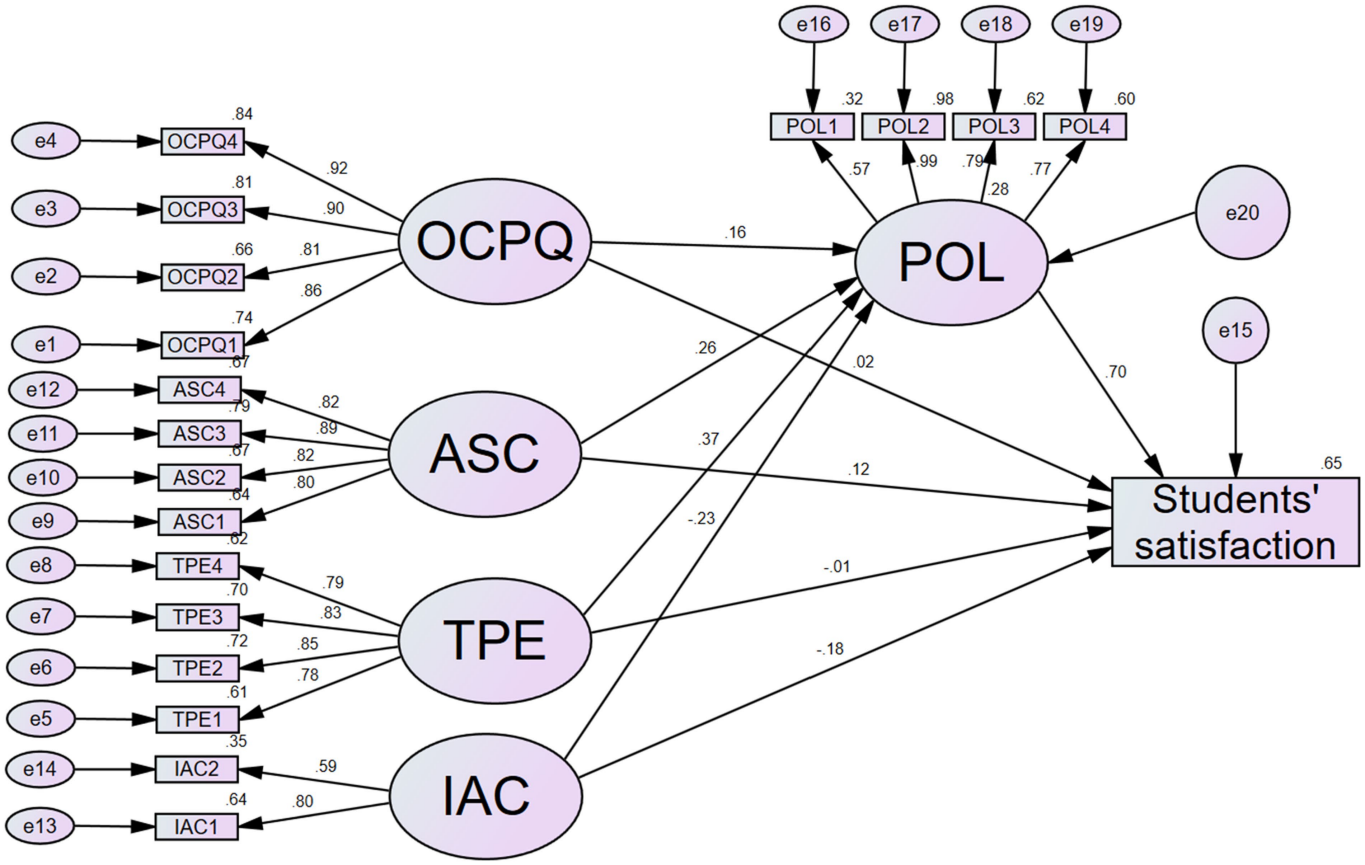
Structural mediation model. Model fit indices: Chi-square (CMIN) = 930.132, degree freedom (df) = 289, relative Chi-square index (CMIN/df) = 3.218, comparative fit index (CFI) = 0.908, standardized root mean square residual (SRMR) = 0.061, root mean square error of approximation (RMSEA) = 0.075. OCPQ, online classroom perceived quality; ASC, acquisition of self-confidence; TPE, teaching performance and engagement; POL, preference for online learning; IAC, internet access and cost.

The structural mediation effect as presented in Figure 4 was examined. SEM in Amos version 26 software was employed to estimate all the direct and indirect paths. The bootstrap method of 5,000 samples at 95% confidence intervals was utilized to establish the mediation effect. According to the rule of thumb for the bootstrapping method, if zero does not fall within the lower and upper bound confidence intervals, then the outcome of the result is significant (Hadi et al., 2016). However, if zero falls within the lower and upper bound confidence intervals, then the outcome of the result is not significant.

The fit statistics for the structural mediation model showed good-fit results with a Chi-square (CMIN) = 930.132, degree freedom (df) = 289, relative Chi-square index (CMIN/df) = 3.218, comparative fit index (CFI) = 0.908, standardized root mean square residual (SRMR) = 0.061, root mean square error of approximation (RMSEA) = 0.075.

The structural mediation path analysis also revealed that all the dimensions of students’ online learning experience had a statistically significant impact on their preference for online learning. The results imply that IAC, TPE, ASC, and OCPQ significantly influenced POL. Hence, H2 (H2a, H2b, H2c, and H2d) are supported. The results also showed that POL had a significant effect on SS; hence, support H3.

Furthermore, the indirect path from IAC to SS through POL had a standardized coefficient value of −0.165 (*p* < 0.025) with a 95% bias-corrected confidence interval (CI) of [−0.223, −0.086]. Since zero did not fall within the 95%CI, it means POL played a significant mediating role in the relationship between IAC and SS. Therefore, the results support H4a. Also, the indirect path coefficient from TPE to SS through POL was 0.259 (*p* < 0.008) with a 95%CI of [0.188, 0.362], supporting H4b. Again, the indirect path coefficient from ASC to SS through POL was 0.185 (*p* < 0.012) with a 95%CI of [0.117, 0.342], supporting H4c. Lastly, the results revealed a standardized indirect path of 0.109 (*p* < 0.006) with a 9%%CI of [0.062, 0.172] from OCPQ to SS through POL, supporting H4d (Table 10, Figure 4).

**Table 10:** Results of structural mediation analysis.

Effects of students’ experience with online-leaning on students’ satisfaction							
	Path	Estimate	S.E.	C.R.		*p*-value	Remarks
H2a	IAC-- > POL	−0.234	0.062	−3.956		***	Supported
H2b	TPE-- > POL	0.368	0.044	7.327		***	Supported
H2c	ASC-- > POL	0.264	0.038	5.755		***	Supported
H2d	OCPQ-- > POL	0.156	0.033	3.678		***	Supported
Effect of preference for online-learning (POL) on students’ satisfaction (SS)							
	Path	Estimate	S.E.	C.R.		*p*-value	Remarks
H3	POL-- > SS	0.703	0.081	12.23		***	Supported
The mediating effect of preference for online learning (POL) in the relationship between students’ experience with online learning and students’ satisfaction							
	Indirect path	Estimate	S.E.	95%CI		*p*-value	Remarks
				LL	UP		
H4a	ICC-- > POL-- > SS	−0.165*	0.041	−0.223	−0.086	0.025	Full mediation
H4b	TPE-- > POL--SS	0.259**	0.051	0.188	0.362	0.008	Partial mediation
H4c	ASC-- > POL-- > SS	0.185*	0.069	0.117	0.342	0.012	Full mediation
H4d	OCPQ-- > POL-- > SS	0.109**	0.036	0.062	0.172	0.006	Partial mediation

## 5. Discussion and conclusion

Student satisfaction is a crucial measure of how well students are doing in their classes and is linked with student retention (Kuo et al., 2013; Fleming et al., 2017) and loyalty to the school (Devinder and Datta, 2003), therefore, educational institutions view student satisfaction as a valuable asset as students are more likely to talk about their experiences positively and return as alumni (Parahoo et al., 2016). A number of studies have outlined the sources of factors that influence student satisfaction, including educational quality, technological features, curriculum and instruction, student characteristics, interaction in classes, learning styles, support services, and on rare occasions, demographic characteristics (Yilmaz, 2017; She et al., 2021). In the phase of the pandemic, most universities, especially in developing countries, started online learning for the first time and had no earlier experience with such a mode of learning, therefore, they were confronted with challenges such as how to adequately engage the students and satisfy their needs in the virtual learning classrooms (Faize and Nawaz, 2020). As a result, several studies have assessed the experience of students during online classes to better ascertain the factors that significantly influence students’ satisfaction and the general success of the online-learning system (Imsa-ard, 2020; Gopal et al., 2021; Ho et al., 2021; Jiang et al., 2021; She et al., 2021; Kornpitack and Sawmong, 2022). In this study, the variables selected to assess the relationship between students’ online learning experience and their satisfaction were online classroom perceived quality, acquisition of self-confidence, teaching performance and engagement, internet access and cost, and preference for online-learning (Fieger, 2012; Unver et al., 2017; Pham et al., 2019; Szopiński and Bachnik, 2021; Segbenya et al., 2022).

We found that teaching performance and engagement positively influence students’ satisfaction. This agrees with a recent study on student satisfaction during the COVID-19 pandemic that showed that there is a significant positive relationship between students’ engagement (interaction) and online learning satisfaction, as well as engagement and acquisition of academic self-efficacy (She et al., 2021). Again, students’ satisfaction is related to their engagement and motivation (Karaoğlan Yılmaz, 2022). Interaction in an online learning setting has been regarded as a critical factor that determines to the extent which students are satisfied with their online education (Cidral et al., 2018). According to Kuo et al. (2014), a high level of interaction with the instructor, other learners, or content leads to high satisfaction and thus represents high engagement in online learning (Kuo et al., 2014). In addition, the quality of the instructor, prompt feedback, course design, and expectation of students positively impact student satisfaction and further, student satisfaction positively impacts students’ performance (Gopal et al., 2021). Invariably, lack of engagement is associated with student dissatisfaction, as insufficient student-teacher interaction and untimely feedback and question-answering from instructors contribute to dissatisfaction, and are among the common challenges students encountered during the first week of online learning during the COVID-19 outbreak (Li et al., 2021). Lack of interaction often leads to poor student engagement and lower student satisfaction (Rahmatpour et al., 2021). Moreover, students who experienced instructors’ poor teaching performance or lack of preparation for courses were dissatisfied with their online learning experience (Li et al., 2021). Quality teaching performance, which includes sufficient interaction in the classroom, adequate student engagement, elaborate course structure, and teacher awareness and facilitation positively influence students’ perceived online learning satisfaction during the pandemic of COVID-19 (Baber, 2020). Therefore, interaction in online learning often translates to students’ engagement in their academic activities, a characteristic of better teaching performance, and positively affects students’ satisfaction (Kim and Kim, 2021).

The perceived quality of online learning significantly influences students’ acceptance or preference for online learning and their overall satisfaction. A study reported that students who perceive a poor formal online-learning orientation tend to respond in higher proportion to problems during online learning, including rejection of online teaching. They also highly associate online learning with insufficient learning resources, untimely feedback and question-answering, poor arrangements and scheduling, and poor preparation of courses (Li et al., 2021), thus, the perceived quality of the online-learning setup significantly positively affects students’ satisfaction with online learning (Li et al., 2021). It is also identified that perceived course quality or ease of use of online learning technology positively influences students’ online learning satisfaction, while computer anxiety negatively shapes students’ satisfaction (Sun et al., 2008). Similarly, students’ perceptions of online learning difficulty influenced their satisfaction during the Covid-19 transition to online education. Students’ satisfaction was negatively affected by perceived technical skill requirements, as it predicted difficulty in using the online learning system and thus, influenced the effective online learning experiences and satisfaction (Conrad et al., 2022). Accordingly to Jiang et al. (2021), perceived quality, ease of use, and usefulness of the online learning platform affect online learning satisfaction in higher education during the COVID-19 pandemic (Jiang et al., 2021). Other studies report factors such as perceived quality, ease of use, the usefulness of online platforms, online learning acceptance or preference, online support service quality, computer self-efficacy, academic self-efficacy, and prior experience as significant influencers of students’ online learning satisfaction (Lee, 2010; Goldstraw et al., 2016; Jiang et al., 2021).

This study found that while internet access and cost negatively influence the overall students’ satisfaction, the acquisition of self-confidence positively influences students’ satisfaction with online learning. This is not surprising since internet connection serves as a critical infrastructural component of e-learning or mobile learning approaches (Delnoij et al., 2020; Korkmaz et al., 2022) but appears to be less accessible and more expensive in developing countries, including Kenya, where only about 35% of the population has access to the Internet (World Bank, 2022). Other reports indicate that a decent internet connection, which is essential for many basic tasks in the COVID-19 era, is out of reach for 90% of people in low-and middle-income countries (Okoth, 2022) and high cost of Internet access remains one of the main barriers to the use of information and communication technology services worldwide (Barton, 2021). Recent studies that examined students’ experience with online learning during the COVID-19 pandemic report that internet connectivity problems, including network congestion, negatively affect student satisfaction (Li et al., 2021; Segbenya et al., 2022) It is also documented that among the key barriers that prevent students from satisfactory online education are accessibility and affordably of Internet usage, in addition to administrative and technical issues, lack of academic and technical skills, interaction, motivation, time, and support for studies (Muilenburg and Berge, 2005). Concerning the positive influence of self-confidence on student satisfaction, similar studies indicate that the acquisition of academic self-efficacy and confidence has a positive effect on students’ engagement within self-directed distance education, where students with high academic self-efficacy and confidence are more engaged in their online studies (Jung and Lee, 2018) and are more likely to experience learning satisfaction (Artino, 2007). Moreover, self-confidence and self-efficacy, which is understood as students’ belief in the capability to perform academically well during an online platform, has been reported to be the most predictive factor of students’ satisfaction (Shen et al., 2013; Jan, 2015). Students’ satisfaction showed a moderate and positive correlation with self-confidence in both simulation-oriented pre-clinical practice and clinical practice among nursing students (Oanh et al., 2021). Other studies have also reported a positive correlation between the levels of students’ self-confidence and their satisfaction, which also positively influences their performance (Almeida et al., 2015; Farrés-Tarafa et al., 2021).

Given the actual situation of the COVID-19 outbreak that impedes traditional face-to-face teaching and learning, online learning serves as a first-line solution for teaching and learning. However, the online learning environment varies profoundly from the traditional classroom situation when it comes to learner satisfaction, motivation, and interaction (Bignoux and Sund, 2018). In this study, approximately 80% of the participants indicated their preference for in-person learning as against online learning, and preference for online learning positively correlated with students’ satisfaction. The less preference could be due to the fact that although the universities were engaging in online classrooms, the issues of preparedness or readiness for online learning, designing, and effectiveness remain challenges to be solved, thus the less preference by students as confirmed by Imsa-ard (2020). Interestingly, some of the challenges that caused the lack of preference for the online-learning included the high cost of internet access and the inability to afford quality devices (Wangkiat, 2021). One possible way to increase online learning preference, acceptance, and satisfaction as demonstrated by Faize and Nawaz (2020) is to identify the problems faced by students during online learning, seek their suggestions for overcoming them and work on the students’ opinions with a team of instructors to modify existing instructional practices during an online class. This results in increased student satisfaction with online learning (Faize and Nawaz, 2020). The abrupt transition to online learning has reportedly contributed to pervasive negative reactions among students (Besser et al., 2022) and has even taken a toll on many students’ mental health (Copeland et al., 2021). Considering these factors among other challenges, it is not surprising that online learning had a low preference rate among university students in Kenya.

### 5.1. Conclusion

According to a report by UNESCO in 2021, more than 220 million students in higher education were affected by the closure of universities in 2020 (UNESCO, 2021). Several studies emphasize the pivotal role that student satisfaction plays in determining the success or failure of online education (Rabin et al., 2019; Gopal et al., 2021). Thus, this study examined the effect of the online-learning experience of higher education students on their satisfaction with online learning in the setting of the COVID-19 pandemic in Kenya. The mediating role of students’ preference on the relationship between online-learning experience and students’ satisfaction was also examined. Regardless of the mass application of online learning in Kenya, approximately 80% of university students still prefer face-to-face classes to online classes. Overall, students indicated a neutral position for the online-leaning experience, implying that although they did not have a better online learning experience, it was at the same time not bad. There is a positive effect of teaching performance and engagement, acquisition of self-confidence, and online classroom perceived quality on students’ satisfaction. On the other hand, students’ satisfaction negatively correlates with internet access and cost. Moreover, students’ preference for online learning positively influences their satisfaction and mediates the relationship between students’ experience and their overall satisfaction. The finding of this study further shed light on the underlying factors that explain students’ online learning satisfaction during the COVID-19 pandemic. Therefore, it provides a guideline for universities and policymakers to make better decisions that enhance students online learning satisfaction and ultimately lead to students’ academic outcomes and achievement.

Limitations of the study include data derived from a relatively short time and a one-time administration of the survey instrument during the academic year. Therefore, the stability of the satisfaction factors over an entire academic year has not been validated. Since the data collection spanned a period of 6 months, the variation in time could also influence the outcome due to the dynamic nature of students’ online learning experience and satisfaction. Moreover, the results best represent the online learning experience and satisfaction in the selected universities in Nairobi and may not necessarily be generalized. Again, online survey research using social media to reach students has the possibility of introducing response bias into the data, making the replication of studies more difficult. Finally, although the investigators collected extensive demographic data on the responding students, there was no possibility of controlling for many of the student characteristics that might have influenced the results. This raises a more general limitation resulting from the ease with which survey instruments can be distributed in the electronic environment. This causes many students to suffer “survey fatigue” which can adversely impact response rates.

## Data availability statement

The raw data supporting the conclusions of this article will be made available by the authors, without undue reservation.

## Ethics statement

Ethical review and approval was not required for the study on human participants in accordance with the local legislation and institutional requirements. The patients/participants provided their written informed consent to participate in this study.

## Author contributions

XL and FO: conceptualization, software, visualization, writing—original draft, writing—review and editing. XL: funding acquisition and project administration. DO: conceptualization, writing—original draft, writing—review and editing. All authors contributed to the article and approved the submitted version.

## Funding

This work was supported by the 2018 Jiangsu provincial key research project related to philosophy and social science in higher educational institutes: Research on Learning Outcomes of Engineering Undergraduates Studying in China from Countries along “One Belt & One Road” (Grant no. 2018SJZDI172), and the teaching reform and research project in Jiangsu University: Research on the Influences of Student-Faculty Interaction upon the Development of Faculty Who Teach International Students Studying in China (Grant no. 2017JGYB010).

## Conflict of interest

The authors declare that the research was conducted in the absence of any commercial or financial relationships that could be construed as a potential conflict of interest.

## Publisher’s note

All claims expressed in this article are solely those of the authors and do not necessarily represent those of their affiliated organizations, or those of the publisher, the editors and the reviewers. Any product that may be evaluated in this article, or claim that may be made by its manufacturer, is not guaranteed or endorsed by the publisher.
