# Supplementary material for: The effect of students’ online learning experience on their satisfaction during the COVID-19 pandemic: The mediating role of preference
Source: Front Psychol. 2023 Jan 31;14:1095073. doi: 10.3389/fpsyg.2023.1095073 (PMC9928211; doi:10.3389/fpsyg.2023.1095073)
Supplement: Supplementary file 1 [file Table_1.DOCX]

Supplementary Material

| Variable | Item code | Items | Reference |
| --- | --- | --- | --- |
| Internet access and cost (IAC) | IAC1 | Internet access is stable and quality in my area | Segbenya et al., (2022) |
|  | IAC2 | Internet access on my device is less costly |  |
| Online classroom perceived-quality (OCPQ) | OCPQ1 | Online classroom learning in my school is quality | Pham et al., (2019) |
|  | OCPQ2 | Online classroom learning is appropriate for my subjects of study |  |
|  | OCPQ3 | The methods and platforms being used are suitability |  |
|  | OCPQ4 | The teaching materials used in online-classroom are motivating and help me to learn |  |
| Teaching performance and engagement (TPE) | TPE1 | The teaching methods used in online-classroom are helpful and effective | Unver et al., (2017); Fieger, (2012) |
|  | TPE2 | The online classroom provides me with a variety of learning materials and activities to promote my learning of the program curriculum |  |
|  | TPE3 | I enjoy how my instructors teach online |  |
|  | TPE4 | There is sufficient teacher-student interaction and engagement |  |
| Acquisition of Self-confidence (ASC) | ASC1 | I am confident that I am mastering the content of the online-classroom activity that my instructors present to me | Unver et al., (2017) |
|  | ASC2 | I am confident that I am developing the skills and obtaining the required knowledge from the online classroom to perform necessary tasks in a real-life setting |  |
|  | ASC3 | My instructors use helpful resources to teach the online-classes |  |
|  | ASC4 | I know how to get help when I do not understand the concepts covered in the online classroom |  |
| Preference for online-learning (POL) | POL1 | I prefer that classes/teaching be done online | Segbenya et al., (2022) |
|  | POL2 | I prefer that quizzes, continuous assessments, and end-of-semester examinations be done online |  |
|  | POL3 | I prefer that supervision of project work/dissertation be done online |  |
|  | POL4 | I prefer that some aspects of practical be done online |  |
| Overall students’ satisfaction (SS) | SS | Overall, I am satisfied with the quality of online classes in my school | Alqurashi, (2019); Fieger, (2012) |

**
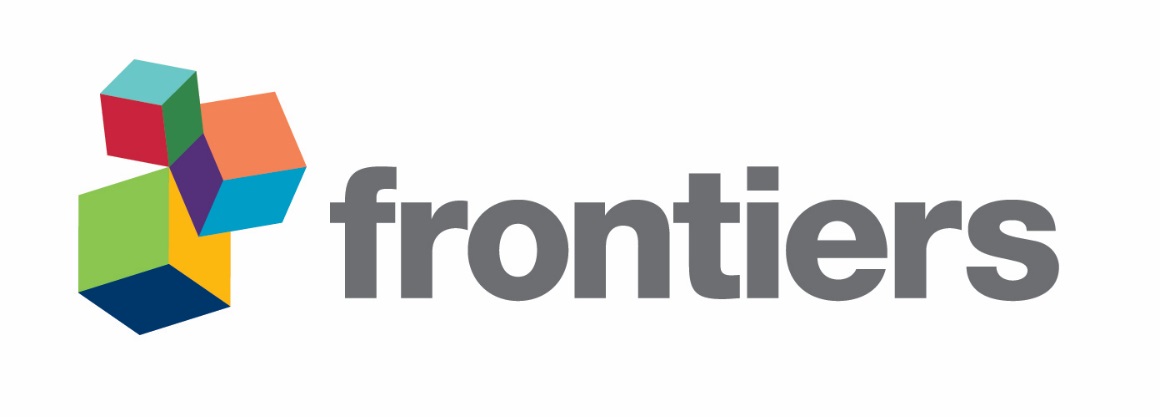
**
